# Three-Year Outcomes of Neovascular Age-Related Macular Degeneration in Eyes That Do Not Develop Macular Atrophy or Subretinal Fibrosis

**DOI:** 10.1167/tvst.10.13.5

**Published:** 2021-11-03

**Authors:** Pierre-Henry Gabrielle, Vuong Nguyen, Jennifer J. Arnold, Sanjeeb Bhandari, Francesco Viola, Odette A. M. Tigchelaar-Besling, Gonzaga Garay-Aramburu, Louise O'Toole, Chui Ming Gemmy Cheung, Daniel Barthelmes, Catherine Creuzot-Garcher, Mark Gillies

**Affiliations:** 1 The University of Sydney, Sydney Medical School, Discipline of Ophthalmology, Save Sight Institute, Sydney, Australia; 2 Department of Ophthalmology, Dijon University Hospital, Dijon, France; 3 Marsden Eye Specialists, Sydney, Australia; 4 Fondazione IRCCS Cà Granda Ospedale Maggiore Policlinico, University of Milan, Milan, Italy; 5 Department of Ophthalmology, Amphia Hospital, Breda, Netherlands; 6 Unidad de Gestión Clínica de Oftalmología, Araba University Hospital, Araba, Spain; 7 Department of Ophthalmology, Mater Private Hospital, Dublin, Ireland; 8 Singapore National Eye Centre, Singapore; 9 Department of Ophthalmology, University Hospital Zurich, University of Zurich, Zurich, Switzerland

**Keywords:** macular atrophy, subretinal fibrosis, age-related macular degeneration, VEGF inhibitors

## Abstract

**Purpose:**

To report the 36-month treatment outcomes of eyes with neovascular age-related macular degeneration (nAMD) receiving vascular endothelial growth factor (VEGF) inhibitors in daily practice who did not develop either subretinal fibrosis (SRFi) or macular atrophy (MA).

**Methods:**

This is a retrospective analysis of data from the Fight Retinal Blindness registry. Treatment-naïve eyes starting intravitreal injection of VEGF inhibitors for nAMD from January 1, 2010, to September 1, 2017, and did not have SRFI and MA at baseline were tracked.

**Results:**

We identified 2478 eligible eyes, of which 1712 eyes did not develop SRFi or MA, 291 developed extrafoveal SRFI or MA, and 475 developed subfoveal SRFi or MA over 36 months. The estimated visual acuity stabilized from 6 months to 36 months in eyes that did not develop SRFI or MA with a mean (95% confidence interval [CI]) change in VA of −1 (−2, 0) letters, whereas eyes that developed extrafoveal (−3 [−5, −2] letters) or subfoveal (−10 [−11, −8] letters) SRFi or MA declined in vision in the same period. Eyes with no or extrafoveal SRFi or MA over 36 months were more likely to maintain their visual improvement from six months to 36 months (odds ratio [OR; 95% CI] = 2.3 [1.5, 3.3] for absence vs. subfoveal SRFi or MA, *P* ≤ 0.01 and OR = 2.0 [1.2, 3.4] for extrafoveal vs. subfoveal MA or SRFi, *P* = 0.01).

**Conclusions:**

Treatment-naïve nAMD eyes receiving VEGF inhibitors maintain their initial six-month visual improvement over three years if they do not develop SRFI or MA.

**Translational Relevance:**

The nAMD is still a major cause of blindness despite antiangiogenic treatments. We found that eyes that did not develop subretinal fibrosis or macular atrophy maintained their initial vision improvement for at least three years, suggesting that identifying treatments for these complications is the final barrier to achieving excellent outcomes in nAMD.

## Introduction

The end-stage sequelae of neovascular age-related macular degeneration (nAMD), macular atrophy (MA), and subretinal fibrosis (SRFi) increase with time, are untreatable, and are associated with poor visual outcomes.^1^^–^^5^ Both clinical trials and observational studies tend to find that visual acuity (VA) improves from baseline for six months after starting treatment with vascular endothelial growth factor (VEGF) inhibitors and then progressively declines thereafter in association with the development of foveal MA or SRFi, with final vision depending mainly on the presenting VA at the start of the treatment and the number of injections.^6^^–^^8^ Older age, presenting VA, and type of choroidal neovascularization (CNV) may predict risk of progression to MA and SRFi under treatment more strongly than treatment strategy and frequency.^1^^,^^9^^,^^10^ Few studies, if any, have investigated whether there is any other mechanism that causes visual loss in eyes with nAMD independently of these features. Here, we tested the hypothesis that eyes with nAMD treated with VEGF inhibitors continue to lose vision through unknown mechanisms, even if they do not develop SRFi or MA.

## Methods

### Design and Setting

This was a retrospective analysis of treatment-naïve eyes that had received intravitreal VEGF inhibitors for nAMD in routine clinical practice tracked in the prospectively designed observational database—The Fight Retinal Blindness! (FRB!) registry. The details of the FRB! database have been previously published.^11^ Analyzed data are 100% completed because all fields must be filled out with in-range values before being accepted by the database. Participants in this analysis included patients from Australia, France, Ireland, Italy, the Netherlands, New Zealand, Singapore, Spain, and Switzerland. Institutional approval was obtained from the University of Sydney, the Royal Australian and New Zealand College of Ophthalmologists, the French Institutional Review Board (IRB) (Société Française d'Ophtalmologie IRB), the Mater Private Hospital IRB in Dublin, Ireland, the Fondazione IRCCS Cà Granda Ospedale Maggiore Policlinico, Milan, Comité de Ética de la Investigación con medicamentos de Euskadi (CEIm-E), and Agencia Española de Medicamentos y Productos Sanitarios (AEMPS), Singhealth Singapore, and the Cantonal Ethics Committee Zurich. All patients gave their informed consent. Informed consent (“opt-in consent”) was sought from patients in France, Ireland, Italy, the Netherlands, Singapore, Spain, and Switzerland. Ethics committees in Australia and New Zealand approved the use of “opt-out” patient consent. This study adhered to the Declaration of Helsinki's tenets and followed the Strengthening the Reporting of Observational Studies in Epidemiology statements for reporting observational studies.^12^

### Data Sources and Measurements

We analyzed data from the nAMD module of the FRB! outcomes registry. Data were obtained from each clinical visit, including the VA, the activity of the underlying choroidal neovascularization (CNV) lesion, the presence of SRFi or MA, treatment given, procedures, and ocular adverse events. Distance VA (uncorrected, corrected and pinhole if required) was measured in Snellen chart and converted as the number of letters read on a logarithm of the minimum angle of resolution (logMAR) VA standard ETDRS chart.^13^ The activity of the CNV lesion was graded by the treating physician based on findings from clinical examination according to a definition provided in the data collection screen from optical coherence tomography (OCT) and dye-based fundus fluorescein angiography, alone or in combination, at each visit. Physician grading of MA and SRFi was implemented in April 2016 into FRB! to comply with the International Consortium for Health Outcomes Measurements (ICHOM) macular degeneration standard set and was recorded prospectively at each visit from then: these data were retrospectively entered for eyes with data entered before this date (n= 245 eyes).^14^ No distinction was made between nonfibrotic scar and fibrotic scar in the grading,^15^ because the diagnosis was based on the concordance between the appearance of SRFi on clinical examination, color fundus photography, and SD-OCT. At each visit, documentation of MA and SRFi was recorded according to the ICHOM standard set as: “Not present” or if present, based on location: “Extrafoveal” or “Subfoveal.”^14^ Repeat treatments were at the physician's discretion in consultation with the patient, thereby reflecting routine clinical practice.

### Patient Selection and Definitions

Treatment-naïve eyes starting intravitreal injection of VEGF inhibitors of either aflibercept (2 mg Eylea; Bayer Healthcare, Leverkusen, Germany), bevacizumab (1.25 mg Avastin; Genetech Inc/Roche, Basel, Switzerland), or ranibizumab (0.5 mg Lucentis; Genetech Inc/Novartis) for nAMD from January 1, 2010, to September 1, 2017, thereby allowing the possibility of having at least 36 months of observations after the initial treatment, and who did not have SRFI or MA at baseline were tracked in the registry. Eyes were excluded if the grading of SRFI or MA was not entered at baseline.

To ensure that eligible eyes did not have SRFI or MA at presentation, the baseline grading of SRFi or MA was based on multimodal imaging at each visit from the start of the treatment to the three-month visit to detect eyes with undiagnosed SRFi or MA at the beginning of the treatment because of intense exudative signs and exclude other reasons of subretinal hyperreflective material such as fibrin or hemorrhage.

Three groups were defined based on the physician grading of SRFi or MA over 36 months of treatment: absence (i.e., eyes that did not develop SRFi or MA graded over 36 months), subfoveal SRFI or MA (i.e., eyes that developed subfoveal SRFi or MA graded over 36 months) and extrafoveal SRFI or MA (i.e., eyes that developed extrafoveal SRFi or MA graded over 36 months). Eye that developed first extrafoveal SRFi or MA and then progressed to subfoveal SRFi or MA over the period were included in the subfoveal SRFI or MA group for the analysis. Eyes that completed at least 1035 days of follow-up were defined as “completers.” Eyes that did not complete 36 months of observations were defined as “non-completers.”

To investigate if vision declined after the initial visual improvement, we analyzed the mean change in VA from six months to 36 months. The initial visual improvement was considered as maintained if the VA change from six months to 36 months was more than −5 letters.

### Outcomes

The main outcome was the estimated mean change in VA from 6 months at 36 months. Secondary visual outcomes included the estimated mean change in VA from baseline to 36 months, the proportion of eyes who maintained vision (VA change > −5 letters), lost ≥10, and ≥15 letters of vision from six months at 36 months, and the mean final VA. Other outcomes of interest were the median time to the development of MA and SRFi over 36 months, the baseline predictors of the development of MA, and SRFi over 36 months, the proportion of visits over 36 months in which the CNV lesion was graded as active, the median time to first grading of CNV inactivity over 36 months, the median time interval between injections over 36 months, the median number of visits and injections administered over 36 months, the rate of noncompletion, the median time and mean VA change to dropout, and the reason for discontinuation over 36 months.

### Statistical Analysis

Descriptive data were summarized using the mean (standard deviation), median (first and third quartiles), and number (percentages) where appropriate. Calculation of crude visual outcomes over 36 months used the last-observation-carried-forward for non-completers.

We compared visual outcomes between SRFi or MA groups over three years using mixed-effects longitudinal generalized additive models with the interaction between the development and location of MA or SRFi during the treatment and time as the main predictor variable. Longitudinal models included all visits from completers and non-completers (all observations until the 36-month visit or dropout). The proportions of eyes who maintained vision, lost 10 letters and 15 letters of vision from six months at 36 months between groups were compared using logistic mixed-effects regression models. Longitudinal and logistic models included age, gender, VA, type of CNV lesion at baseline and lens status during the follow-up, and nesting of outcomes within practitioners and patients with bilateral disease as random effects. Generalized Poisson regression models were used to compare the number of injections and visits between groups over 36 months. Longitudinal, logistic and generalized Poisson models included age, gender, VA, type of CNV lesion at baseline and lens status during the follow-up, and nesting of outcomes within practitioners and patients with bilateral disease as random effects. Cox-proportional hazards models and Kaplan-Meier survival curves were used to assess and visualize the time to first grading of inactivity, first grading of SRFi and MA, and non-completion rates over 36 months.

A *P* value = 0.05 was considered statistically significant. All analyses were conducted using R software version 4.0.2 (R Project for Statistical Computing, Vienna, Austria; R Foundation for Statistical Computing; 2020, https://cran.rproject.org) with the *mgcv* package (V1.8-31) for the generalized additive (mixed) model computation and the *survival* package (V3.1-11) for the time to first CNV inactivity, development of SRFi and MA and drop-out analysis.

## Results

We identified 2478 treatment-naïve eyes from 2218 patients who were eligible for the present analysis. The number of eyes at each selection criterion is shown in Supplementary Figure S1. There were 1712, 291, and 475 eyes that did not develop, developed extrafoveal, and developed subfoveal SRFi or MA, respectively, over 36 months. The mean (standard deviation [SD]) presenting VA in the subfoveal SRFi or MA group was significantly worse than extrafoveal and no SRFI or MA groups (51 [22] vs. 62 [16] vs. 63 [17] letters, respectively; *P* < 0.01) with significantly fewer eyes with VA ≥ 70 letters and more eyes with VA ≤ 35 letters (Table 1).

**Table 1.** Baseline Characteristics of the Study Population

		Macular Atrophy or Subretinal Fibrosis			
		Developed Over 36 Months of Treatment			
	Overall	Absent	Extrafoveal	Subfoveal	*P*
Eyes	2478	1712	291	475	
Patients	2218	1586	268	452	
Females, %	61	61	66	61	0.23
Age, mean (SD)	78 (9)	78 (9)	78 (9)	79 (9)	**0.013**
Lens status (phakic), %	77	79	74	70	**<0.01**
Visual acuity, mean (SD)	61 (18)	63 (17)	62 (16)	51 (22)	**<0.01**
≥70 letters, n (%)	996 (40)	777 (45)	108 (37)	111 (23)	**<0.01**
≤35 letters, n (%)	271 (11)	132 (8)	24 (8)	115 (24)	**<0.01**
Angiographic lesion size, median µm (Q1, Q3) ^a^	1800 (1000, 3000)	1900 (1000, 3000)	1760 (1000, 2600)	1500 (601, 2500)	**0.022**
Angiographic lesion type, % ^a^					**<0.01**
Type 1	37	39	41	28	
Type 2	14	13	16	17	
Type 3	5	5	11	4	
Polypoidal choroidal vasculopathy	7	8	8	4	
Peripapillary choroidal neovascularization	1	2	1	1	
Unavailable	35	33	23	46	
Type of VEGF inhibitors, %					**<0.01**
Aflibercept	39	36	47	42	
Bevacizumab	28	28	28	26	
Ranibizumab	34	36	25	32	

Q1, first quartile; Q3, third quartile.

a Missing values: n = 1068 eyes (angiographic lesion size), n = 856 eyes (angiographic lesion type).

Significant *P* values are highlighted in bold.

### Visual Outcomes Over Three Years

The 36-month treatment outcomes of the study population are reported in Table 2. The overall crude mean (95% confidence interval [95%CI]) change in VA improved by 6 (5, 6) letters from baseline to six months (mean [SD] six-month VA = 66 [17] letters) and did not differ significantly between groups (*P* = 0.14) (Table 2). Subsequently, the overall mean change in VA progressively dropped by −2 (−3, −2) letters from 6 months to 36 months (mean [SD] final VA = 64 [20] letters).

**Table 2.** Thirty-Six–Month Visual Outcomes of the Study Population and by Subgroup According to the Development of Macular Atrophy or Subretinal Fibrosis

					Adjusted Group Comparison					
		MA or SRFi During Treatment			Absent vs. Extrafoveal		Absent vs. Subfoveal		Extrafoveal vs. Subfoveal	
	All Eyes	Absent	Extrafoveal	Subfoveal	OR, mean difference or ratios (95% CI)	*P*	OR, mean difference or ratios (95% CI)	*P*	OR, mean difference or ratios (95% CI)	*P*
Eyes, n	2478	1712	291	475						
Patients, n	2218	1586	268	452						
Visual outcomes (completers and non-completers)										
Baseline VA letters, mean (SD)	61 (18)	63 (17)	62 (16)	51 (22)						
VA at 6 months letters, mean (SD)	66 (17)	69 (15)	69 (13)	56 (21)						
Final VA, mean (SD)	64 (20)	68 (17)	65 (18)	48 (26)						
Crude VA change from baseline to 6 months letters, mean (95% CI)	+6 (5, 6)	+6 (5, 6)	+7 (5, 8)	+5 (3, 6)						
Crude VA change at 36 months letters, mean (95% CI) ^a^										
From baseline	+3 (2, 4)	+5 (4, 5)	+3 (1, 6)	−4 (−6, −1)						
From 6 months	−2 (−3, −2)	−1 (−1, 0)	−4 (−5, −2)	−8 (−10, −6)						
Estimated VA change at 36 months letters, mean (95% CI) ^b^										
From baseline	+2 (1, 3)	+6 (5, 6)	+4 (2, 5)	−9 (−10, −7)	+2 (0, 4)	0.20	+14 (13, 16)	**<0.01**	+12 (10, 14)	**<0.01**
From 6 months	−4 (−5, −3)	−1 (−2, 0)	−3 (−5, −2)	−10 (−11, −8)	+2 (1, 4)	0.32	+9 (7, 10)	**<0.01**	+6 (4, 8)	**<0.01**
VA maintained from 6 months at 36 months (VA change > -5 letters), % ^c^	74	79	69	58	1.1 (0.7, 1.7)	0.55	2.3 (1.5, 3.3)	**<0.01**	2.0 (1.2, 3.4)	**<0.01**
VA loss from 6 months at 36 months ≥ 10 letters, % ^c^	19	14	22	38	0.7 (0.4, 1.1)	0.09	0.3 (0.2, 0.5)	**<0.01**	0.4 (0.2, 0.7)	**<0.01**
VA loss from 6 months at 36 months ≥ 15 letters, % ^c^	13	8	16	29	0.5 (0.3, 0.9)	0.08	0.2 (0.1, 0.4)	**<0.01**	0.4 (0.2, 0.8)	**<0.01**
VA ≥70 letters (baseline/6 months/36 months), %	40/57/54	45/63/62	37/62/54	23/32/27						
VA ≤35 letters (baseline/6 months/36 months), %	40/7/12	45/5/6	37/3/10	23/18/35						
Injections and visits outcomes (completers only)										
Completer eyes, n	1353	852	205	296						
Completer patients, n	1217	789	190	283						
Number of injections at 36 months, median (Q1, Q3) ^d^	19 (14, 25)	20 (14, 26)	18 (14, 23)	18 (12, 23)	1.1 (0.9, 1.2)	0.07	1.1 (1.0, 1.1)	0.25	1.0 (0.9, 1.1)	0.80
Injections yearly first year, median (Q1, Q3) ^d^	8 (6, 10)	8 (6, 10)	8 (6, 9)	8 (6, 10)	1.0 (1.0, 1.1)	0.17	1.0 (1.0, 1.1)	1.0	0.9 (0.9, 1.0)	0.29
Injections yearly second year, median (Q1, Q3) ^d^	6 (3, 8)	6 (4, 8)	5 (3, 8)	5 (3, 8)	1.1 (1.0, 1.2)	0.27	1.1 (0.9, 1.2)	0.46	1.0 (0.9, 1.1)	0.91
Injections yearly third year, median (Q1, Q3) ^d^	5 (3, 8)	6 (4, 8)	5 (3, 6)	5 (2, 7)	1.2 (1.0, 1.3)	**0.011**	1.1 (1.0, 1.3)	**0.012**	1.0 (0.9, 1.2)	0.96
Time between injections days over 36 months, median (Q1, Q3)^e^	48 (36, 63)	45 (35, 63)	55 (42, 67)	49 (36, 63)	−3 (−7, 1)	0.21	+1 (−3, 5)	0.49	+4 (−1, 9)	0.18
Number of visits at 36 months, median (Q1, Q3) ^d^	25 (20, 32)	26 (20, 33)	24 (19, 31)	24 (19, 30)	1.0 (0.9, 1.1)	0.06	1.0 (1.0, 1.1)	0.28	1.0 (0.9, 1.1)	0.72
Visits yearly first year, median (Q1, Q3) ^d^	11 (9, 13)	11 (9, 13)	11 (9, 13)	11 (9, 13)	1.0 (1.0, 1.1)	0.20	1.0 (0.9, 1.0)	0.85	1.0 (0.9, 1.0)	0.15
Visits yearly second year, median (Q1, Q3) ^d^	8 (5, 10)	8 (5, 10)	7 (5, 10)	7 (5, 9)	1.0 (1.0, 1.1)	0.37	1.0 (1.0, 1.1)	0.58	1.0 (0.9, 1.1)	0.93
Visits yearly third year, median (Q1, Q3) ^d^	7 (5, 10)	7 (5, 10)	7 (4, 9)	6 (4, 9)	1.1 (1.0, 1.2)	**<0.01**	1.1 (1.0, 1.2)	**<0.01**	1.0 (0.9, 1.1)	0.98

Q1, first quartile; Q3, third quartile.

a Last observation carried forward for non-completers.

b Calculated from adjusted non-linear mixed-effects regression models.

c Odds ratios (95% CI) were calculated from adjusted logistic mixed-effects regression models.

d Adjusted ratio (95% CI) of number of injections or number of injections yearly or visits between groups. It was calculated from adjusted generalized Poisson regression models.

e Mean differences (95% CI) were calculated from adjusted linear mixed-effects regression models.

Pairwise comparison with Holm-Bonferroni adjustment was used for multiple comparisons. Significant p-values are highlighted in bold.

**Figure 1.**
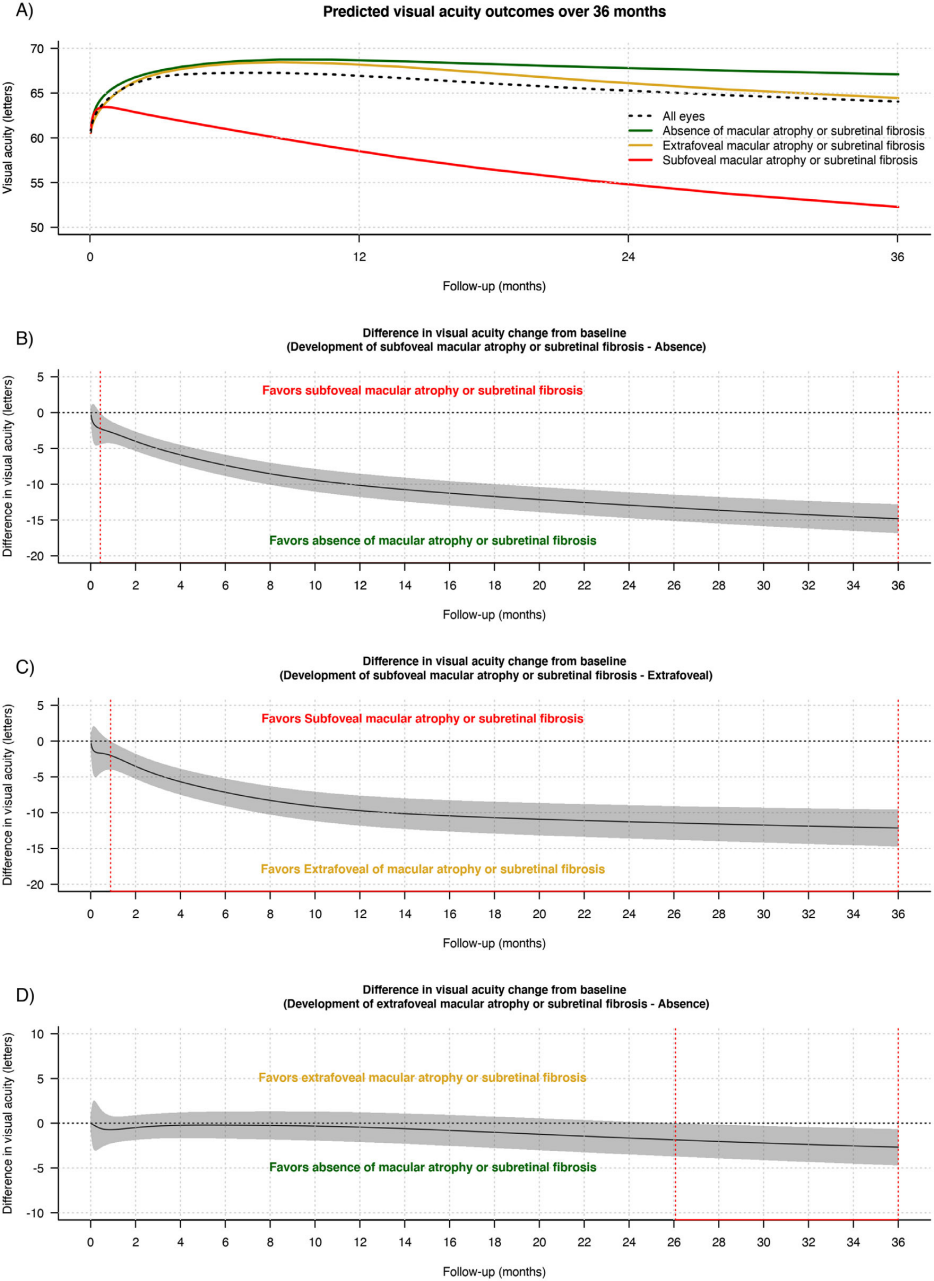
Line graphs showing the mean predicted VA (*solid lines*) in logMAR letters with time (A) in all eyes (*black dashed line*) and depending on the development of MA or SRFi and its location during treatment (absence [*green*], extrafoveal [*gold*], and subfoveal [*red*]) and the difference in the mean change in VA from baseline between the different subgroups of SRFi or MA (B, C and D) over 36 months irrespective of whether eyes completed or did not complete 36 months of observations from starting treatment. Predictions were made from a generalized additive model for all eyes. In B, C, and D, the *gray shaded area* represents the 95% CI, and the *red dashed lines* indicate areas where the 95% CI does not intersect with 0.

Figure 1 shows the estimated mean VA from longitudinal models in all eyes (including completers and non-completers) and according to the development of SRFi or MA and its foveal involvement. Overall, the estimated mean (95%CI) change in VA was 2 (1, 3) letters from baseline to 36 months (Table 2 and Fig. 1A). The difference in the estimated mean (95% CI) in VA change from baseline at 36 months was significantly in favor of eyes with absence or extrafoveal SRFi or MA than eyes with subfoveal SRFi or MA (14 [13, 16] letters for absence vs. subfoveal, *P* < 0.01; 12 [10, 14] letters for extrafoveal vs. subfoveal, *P* < 0.01) (Table 2 and Figs. 1B and 1C). Overall, the estimated mean (95% CI) change in VA from six months at 36 months was −4 (−5, −3) letters (Table 2). There was a clinically significant drop in the estimated VA from 6 months at 36 months in eyes developing extrafoveal or subfoveal SRFi or MA over 36 months with −3 (−5, −2) and −10 (−11, −8) letters, respectively; while the estimated VA stabilized from six months to 36 months in eyes that did not develop SRFI or GA with −1 (−2, 0) letters at 36 months (Table 2, Fig. 1D).

Eyes with no or extrafoveal SRFi or MA over 36 months were more likely to maintain their visual improvement from 6 months to 36 months (odds ratio OR [95% CI] = 2.3 [1.5, 3.3] for absence vs. subfoveal SRFi or MA, *P* ≤ 0.01 and OR = 2.0 [1.2, 3.4] for extrafoveal vs. subfoveal MA or SRFi, *P* = 0.01) (Table 2). Those eyes were also less likely to lose ≥10 and ≥15 letters of vision from 6 months to 36 months than eyes that developed subfoveal SRFi or MA over 36 months (Table 2). No difference was found between eyes with no SRFi or MA and those that developed extrafoveal SRFi or MA (Table 2).

One hundred sixty-nine eyes underwent cataract extraction, and 15 eyes underwent vitrectomy over three years of treatment. Eyes that did not develop SRFi or MA and developed extrafoveal SRFi or MA tended to have better visual outcomes if they had cataract surgery during the 36 months treatment (See Supplementary Table S1). Crude mean VA change from baseline (−1 [−17, 14] letters vs. 3 [2, 4] letters; *P* = 0.38) and from six months (−5 [−23, 12] letters vs. −3 [−3, −2] letters; *P* = 0.50) at 36 months was similar between eyes that had vitrectomy or not during treatment, respectively.

### Subretinal Fibrosis or Macular Atrophy Development Over Three Years

**Figure 2.**
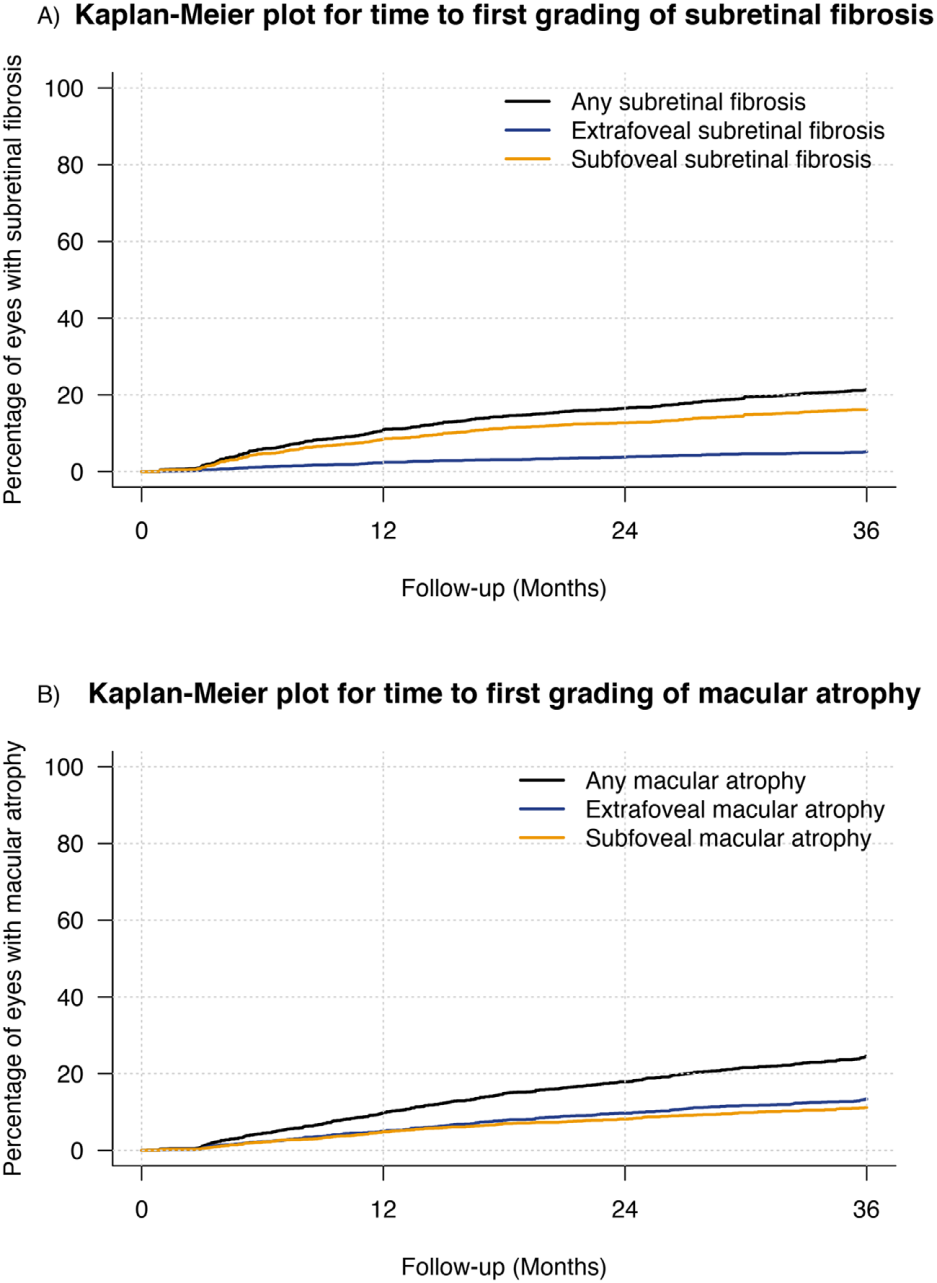
Kaplan-Meier plots for time from starting treatment to (A) the development of macular atrophy and (B) subretinal fibrosis of any location (*black*) and according to its location (extrafoveal [*royal blue*] and subfoveal [*orange*]) over 36 months.

Kaplan-Meier estimates the cumulative rate of SRFi and MA and according to their foveal involvement over three years of treatment (Fig. 2). The cumulative rate of SRFi was 11% (8.5% subfoveal vs. 2.5% extrafoveal) at 12 months, 16.5% (12.7% subfoveal, 3.8% extrafoveal) at 24 months, and 21.3% (16.2% subfoveal, 5.2% extrafoveal) at 36 months (Fig. 2A). The cumulative rate of MA was 9.9% (4.9% subfoveal, 5% extrafoveal) at 12 months, 17.9% (8.2% subfoveal, 9.7% extrafoveal) at 24 months, and 24.5% (11.1% subfoveal, 13.4% extrafoveal) at 36 months (Fig. 2B). Eyes with worse baseline VA and type 2 CNV were more likely to develop SRFi over 36 months (hazard ratio [HR] = 0.76 [0.70, 0.83] every 10 letters of increase in baseline VA, *P* < 0.01 and HR = 1.5 [1.2, 1.8] for type 2 vs. type 1 CNV, *P* = 0.05 [Tukey adjusted P = 0.047 for multiple comparisons]). Older patients and eyes with worse baseline VA and baseline Type 3 CNV were more likely to develop MA over 36 months of treatment (HR = 1.29 [1.14, 1.45] every 10 years of increase in presenting age, *P* < 0.01; HR = 0.82 [0.76, 0.89] every 10 letters of increase in baseline VA, *P* < 0.01; HR = 1.9 [1.5, 2.4] for type 3 vs. type 1 CNV, *P* = 0.021 [Tukey adjusted *P* = 0.018]). That is, the incremental likelihood of developing MA over 36 months increased by 18% every 10 ETDRS letters worse the baseline vision and by 29% each additional 10 years in age at the start of the treatment.

### Choroidal Neovascularization Activity Outcomes Over Three Years

**Figure 3.**
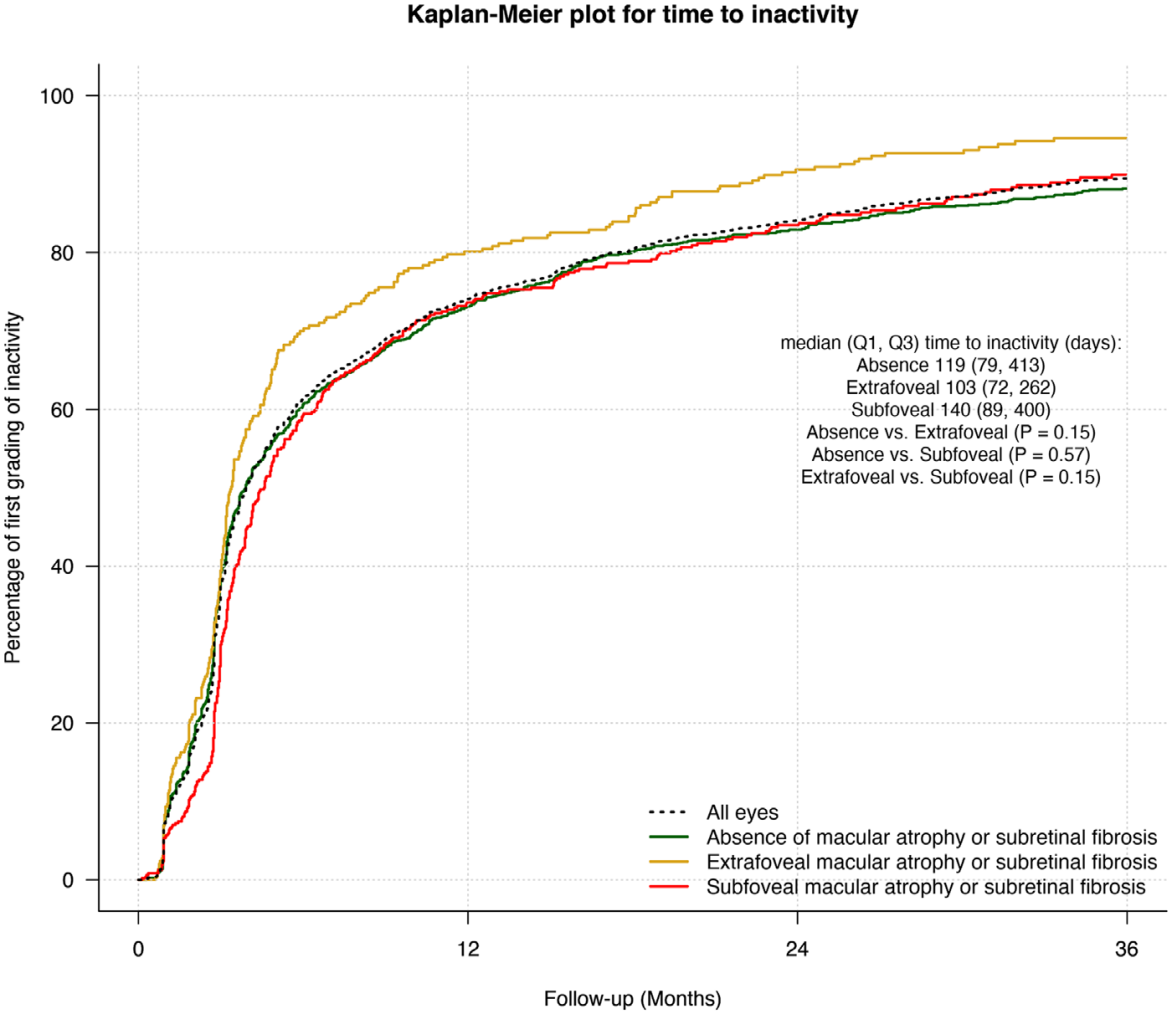
Kaplan-Meier plots for time from starting treatment to the first grading of inactivity in all eyes (*black dashed line*) and depending on the development of macular atrophy or subretinal fibrosis and its location during treatment (absence [*green*], extrafoveal [*gold*], and subfoveal [*red*]) over 36 months.

Overall, the proportion of active visits in eyes completing 36 months was 55%, lower in eyes that developed extrafoveal SRFi or MA (43.3%) than eyes that did not develop (56%) or developed subfoveal SRFI or MA (56%) (*P* = 0.044). The median (Q1, Q3) time to first grading of inactivity was 119 (82, 385) days and was not significantly different between subgroups (Fig. 3).

### Injections and Visits Over Three Years

The median (Q1, Q3) number of injections was 19 (14, 25) over three years in completers with 8 (6, 10), 6 (3, 8) and 5 (3, 8) median (Q1, Q3) injections yearly at first, second, and third year, respectively (Table 2). The adjusted ratio (95% CI) of the number of injections and visits from the generalized Poisson regression model was similar between subgroups according to the development of SRFi or MA in eyes completing 36 months (Table 2). Eyes that did not develop SRFi or MA tended to be more aggressively treated (adjusted ratio third-year injection yearly [95% CI] = 1.2 [1.0, 1.3] for absent vs. extrafoveal, *P* = 0.011 and 1.1 [1.0, 1.3] for absent vs. subfoveal, *P* = 0.012) and monitored (adjusted ratio third-year visit yearly [95% CI] = 1.1 [1.0, 1.2] for absent vs. extrafoveal, *P* < 0.01 and 1.1 [1.0, 1.2] for absent vs. subfoveal, *P* < 0.01) during the third year than eyes that developed extrafoveal and subfoveal SRFi or MA over 36 months of treatment (Table 2).

### Outcomes of Eyes not Completing Three Years

The overall non-completion rate over 36 months was 45.4% (1125 eyes) and was more frequent in eyes that did not develop SRFi or MA than eyes that developed extrafoveal or subfoveal SRFi or MA (50% absence vs. 30% extrafoveal vs. 38% subfoveal, *P* < 0.01; see Supplementary Fig. S2). The mean VA at drop out was significantly better than the mean baseline VA each year of drop out (Supplementary Fig. S3). The reasons for patients discontinuing treatment were tracked in 141 (14%) eyes. These were mainly not related to a poor outcome (71%, 100 eyes): treatment considered as successful 39% (54 eyes), patient transferred to another doctor 14% (20 eyes), death 14% (20 eyes), and medical contraindication 4% (six eyes).

## Discussion

The present study reports that treatment-naïve nAMD eyes receiving VEGF inhibitors maintain their initial six-month visual improvement over three years of treatment in routine clinical practice if they do not develop SRFI or MA. This is not necessarily surprising, because SRFi and MA are well-known associations of poor long-term visual outcomes in treated nAMD eyes.^2^^,^^3^^,^^5^^,^^7^^,^^15^^–^^18^ The significance of our findings is that there is probably no other disease process that causes loss of the initial gains seen in eyes treated for nAMD. Although it would be helpful for this finding to be consolidated and extended in future studies, it appears that the prevention of MA and SRFi is the final obstacle to achieving better, enduring outcomes in nAMD.

Not surprisingly, eyes in the three groups were not comparable at baseline particularly regarding presenting VA. This may be possibly due to an increased amount of blood or fibrin at baseline in the incident SRFI or MA group. We tried to limit the inclusion of baseline SRFi or MA eyes in the study using multimodal imaging definition to differentiate these features with other causes of subretinal hyperreflective material and defined the status of baseline SRFi or MA on the first three months treatment visits.

Our results emphasize that the development or extension of MA or SRFi in the subfoveal region is associated with poor long-term visual outcomes.^15^^–^^19^ Eyes that developed subfoveal SRFi or MA over 36 months had at least two to three lines difference in the final estimated mean VA change from baseline, were at least half as likely to maintain six-month visual improvement at 36 months and twice as likely to have a two-line or three-line VA loss from six months at 36 months than eyes that developed extrafoveal SRFi or MA over 36 months of treatment.

Approximately 20% and 25% of eligible eyes developed SRFI and MA respectively by 36 months from the start of the treatment. These cumulative rates of SRFi and MA were similar to those reported elsewhere.^9^^,^^16^^,^^17^ The Macular atrophy in Pro re Nata versus Treat-and-Extend (MANEX) study reported that the incidence of new atrophy lesion in consecutive naïve treated nAMD eyes receiving VEGF inhibitors was approximately 19% and 22% at 2 and 3 years of treatment, respectively.^9^ In the comparison of AMD Treatments Trials (CATT), non-geographic atrophy and scar rates were estimated to be approximately 12 to 19% and 16 to 20% at 2 years, respectively, depending on the type of drug and treatment pattern.^17^^,^^18^ The FRB registry has implemented the ICHOM classification grading of SRFi and MA to standardize the diagnosis of these features and compare outcomes between different reports. Our results are derived from an extensive observational database with multiple practitioners grading the clinical features, which may be less precise than in reports from RCTs such as CATT.^16^^–^^18^ However, most of the practitioners contributing data are retina specialists who have agreed to use the ICHOM multimodal grading definition of these features. These real-world findings also reflect how diagnosis and treatment decision would be made in daily clinical practice if an effective drug were developed for preventing or treating MA or SRFi.

The three-year visual real-world outcomes of VEGF inhibitors for nAMD were reasonably good (mean +3 letters improvement from baseline) with a median number of injections yearly of eight, six, and five during the first, second, and third year of treatment. Previous retrospective observational studies have reported poorer outcomes at three years.^20^^–^^23^ It is difficult to compare our study to earlier reports because we included only eyes that had been diagnosed early without SRFi or MA when they started treatment.

There was no difference in treatment and visits frequency between the subgroups. However, we found that eyes with no SRFi or MA, which achieved the best visual outcomes, were more aggressively treated and monitored during the third year of treatment. This reinforces the idea that initial VA improvement in nAMD can be maintained with more intensive or proactive treatment approaches in clinical practice.^8^

As previously reported in the literature, presenting VA was a significant predictive factor of MA and SRFi development in our study.^1^^–^^3^ Type 3 CNV was associated with an increased risk of MA and type 2 CNV with an increased risk of SRFi, which has been confirmed in previous reports.^1^^,^^9^^,^^10^^,^^19^

Loss to follow-up may introduce bias because eyes that discontinue may drop out because of poor outcomes or sometimes because of good response to treatment and stabilization of vision. The rate of non-completion was, in fact, highest in eyes that did not develop SRFi or MA over 36 months of treatment. The mean VA of the eyes that dropped out tended to be better than the presenting VA when treatment had discontinued, suggesting that those eyes tended to have good visual outcomes. Most (70%) of tracked reasons for discontinuation were mainly not related to a poor outcome. Our estimated outcomes, particularly in eyes that did not develop SRFi or MA over the study, may be inferior to the actual results if patients with good vision tended to discontinue follow-up within 36 months.

We acknowledge several limitations that are mostly inherent in retrospective observational studies. Injection decisions in routine clinical practice are made without a guided management protocol, so they may vary among retinal specialists compared to RCTs. The grading of SRFi, MA, lesion type, and lesion activity may have interphysician variability. The FRB! registry receives data from a wide variety of international practices and practitioners. Thus we believe our data are fairly representative of clinicians worldwide, which may reduce potential bias caused by this variability to some extent. We also included nesting of outcomes within practitioners in our models to help account for these effects. This analysis's main strengths are its originality and the large number of eyes that were studied over a long time period in daily clinical practice.

To conclude, our study suggests that SRFi and MA are the main retinal causes of the long-term (three-year) visual decline in vision in nAMD eyes in routine clinical practice. Early diagnosis and appropriate application of treatment regimens to prevent these features and their extension to the subfoveal region stabilize the visual improvement after the start of the treatment. Further research is warranted to determine whether these findings hold for longer periods. There is a need to develop new drugs with potential antifibrotic and neuroprotective effects, combined with VEGF inhibitors, to prevent or even treat these end-stage features of nAMD and further improve visual outcomes and the quality of life of our patients.

## Supplementary Material

Supplement 1

Supplement 2

Supplement 3

Supplement 4
