# Supplementary figures and images for: Three-Year Outcomes of Neovascular Age-Related Macular Degeneration in Eyes That Do Not Develop Macular Atrophy or Subretinal Fibrosis
Source: Transl Vis Sci Technol. 2021 Nov 3;10(13):5. doi: 10.1167/tvst.10.13.5 (PMC8572511; doi:10.1167/tvst.10.13.5)

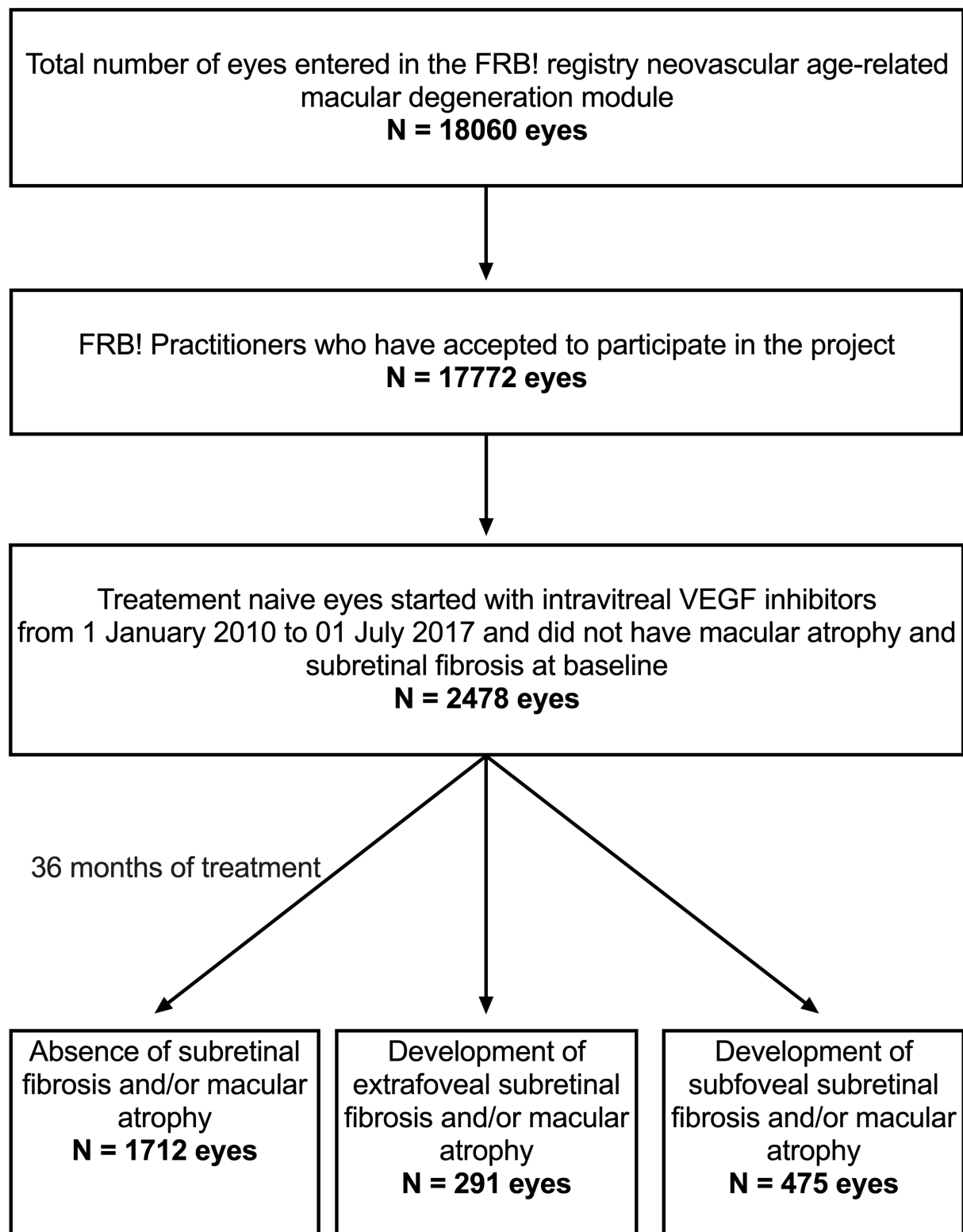

**Figure S1.** Flow chart showing the number of eyes remaining at each selection criterion.

Supplement: Supplement 1 [file tvst-10-13-5_s001.pdf]
