# Supplementary material for: Three-Year Outcomes of Neovascular Age-Related Macular Degeneration in Eyes That Do Not Develop Macular Atrophy or Subretinal Fibrosis
Source: Transl Vis Sci Technol. 2021 Nov 3;10(13):5. doi: 10.1167/tvst.10.13.5 (PMC8572511; doi:10.1167/tvst.10.13.5)
Supplement: Supplement 2 [file tvst-10-13-5_s002.pdf]

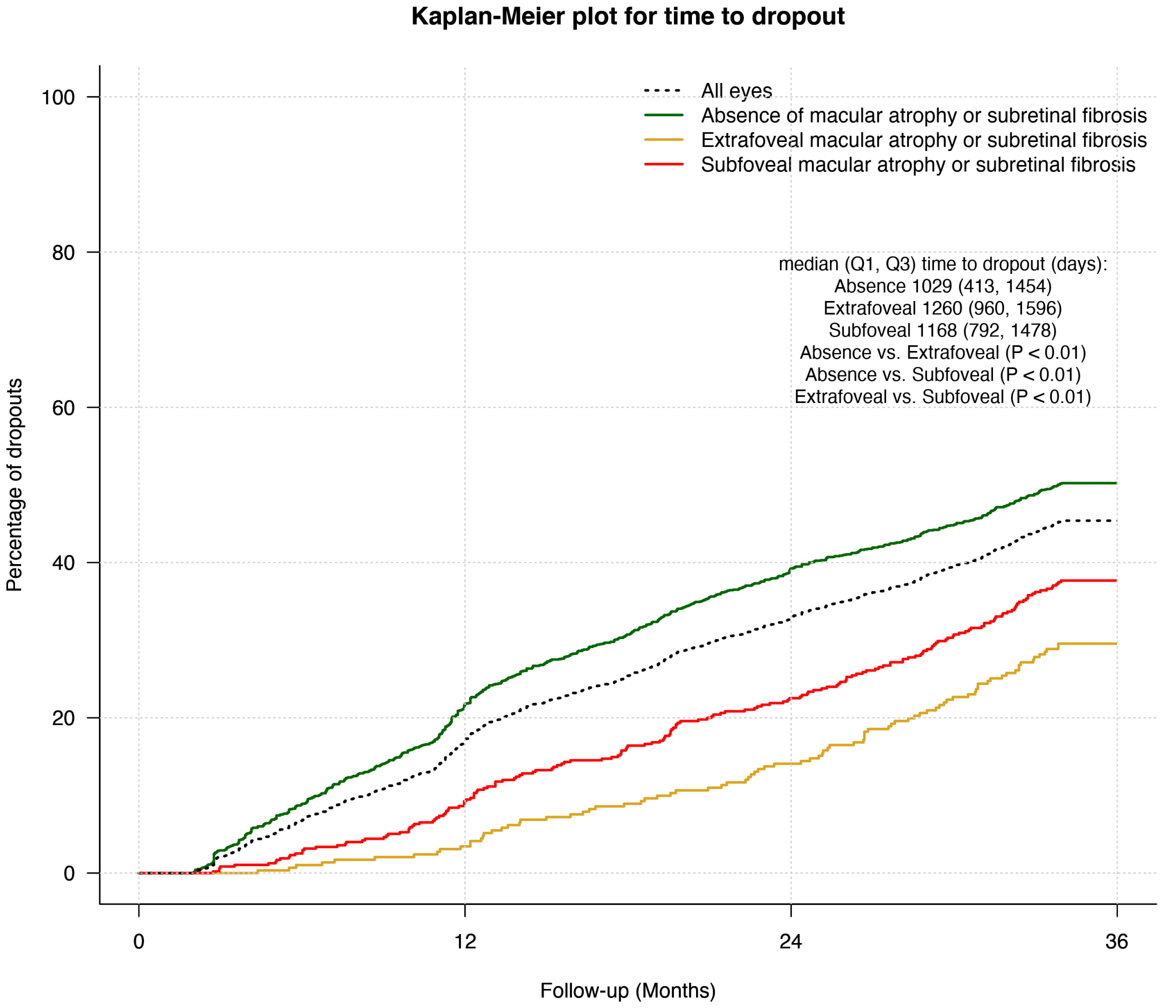

Figure S2. Kaplan-Meier plots for time from starting treatment to drop-out in all eyes (black dashed line) and according to the development of macular atrophy and/or subretinal fibrosis and its location during treatment (absence [green], extrafoveal [gold] and subfoveal [red]) over 36 months.
