# Supplementary material for: Three-Year Outcomes of Neovascular Age-Related Macular Degeneration in Eyes That Do Not Develop Macular Atrophy or Subretinal Fibrosis
Source: Transl Vis Sci Technol. 2021 Nov 3;10(13):5. doi: 10.1167/tvst.10.13.5 (PMC8572511; doi:10.1167/tvst.10.13.5)
Supplement: Supplement 3 [file tvst-10-13-5_s003.pdf]

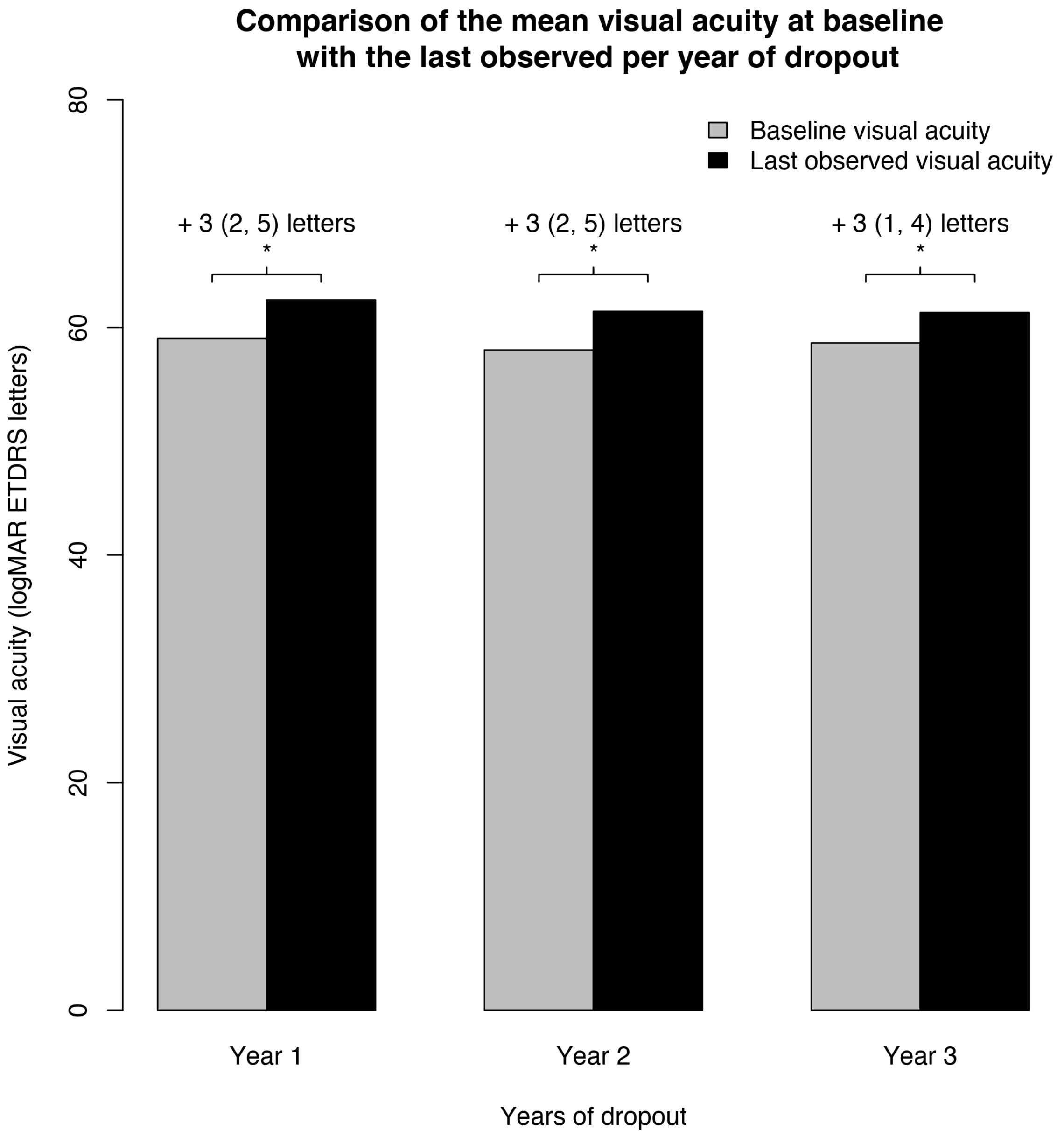

Figure S3. Comparison of the mean visual acuity in LogMAR letters at baseline (grey) with the last observed mean visual acuity (black) in eyes of patients who dropped out each year of follow-up.
