# Supplementary material for: Three-Year Outcomes of Neovascular Age-Related Macular Degeneration in Eyes That Do Not Develop Macular Atrophy or Subretinal Fibrosis
Source: Transl Vis Sci Technol. 2021 Nov 3;10(13):5. doi: 10.1167/tvst.10.13.5 (PMC8572511; doi:10.1167/tvst.10.13.5)
Supplement: Supplement 4 [file tvst-10-13-5_s004.pdf]

Table S1. 36-month visual outcomes stratified by cataract extraction and the development of macular atrophy (MA) and/or subretinal fibrosis (SRFi) and its location in baseline phakic eyes.

	36 months of follow-up of eyes **without** subretinal fibrosis and/or macular atrophy		
	No cataract extraction	Cataract extraction	P
Eyes	1248	100	
Patients	1163	95	
Time to cataract extraction days, median (Q1, Q3)	-	541 (300, 798)	-
Baseline VA letters, mean (SD)	63 (17)	60 (16)	**<0.01**
6 months VA letters, mean (SD)	69 (15)	64 (16)	**<0.01**
Crude VA change from baseline to 6 months letters, mean (95% CI)	+6 (5, 6)	+4 (2, 7)	0.49
Final VA, mean (SD)	68 (17)	71 (11)	**0.020**
Crude VA change at 36 months letters, mean (95% CI) [a]			
From baseline	+4 (3, 5)	+12 (9, 15)	**<0.01**
From 6 months	-1 (-2, -1)	+7 (4, 10)	**<0.01**
VA maintained from 6 months at 36 months (VA change ≥ -5 letters), %	79	88	**0.03**
VA loss from 6 months at 36 months ≥ 10 letters, %	14	7	0.06
VA loss from 6 months at 36 months ≥ 15 letters, %	8	2	**0.04**

	36 months of follow-up of eyes with **subfoveal** subretinal fibrosis and/or macular atrophy		
	No cataract extraction	Cataract extraction	P
Eyes	288	44	
Patients	278	42	
Time to cataract extraction days, median (Q1, Q3)	-	553 (372, 816)	
Baseline VA letters, mean (SD)	52 (22)	47 (26)	0.23
6 months VA letters, mean (SD)	57 (22)	52 (22)	0.19
Crude VA change from baseline to 6 months letters, mean (95% CI)	+5 (3, 7)	+5 (-1, 11)	0.93
Final VA, mean (SD)	49 (25)	46 (29)	0.41
Crude VA change at 36 months letters, mean (95% CI) [a]			
From baseline	-2 (-5, 1)	-1 (-9, 6)	0.81

From 6 months	-7 (-10, -5)	-6 (-14, 2)	0.72
VA maintained from 6 months at 36 months (VA change ≥ -5 letters), %	61	63	0.64
VA loss from 6 months at 36 months ≥ 10 letters, %	35	36	0.95
VA loss from 6 months at 36 months ≥ 15 letters, %	27	34	0.40

	36 months of follow-up of eyes with **extrafoveal** subretinal fibrosis and/or macular atrophy		
	No cataract extraction	Cataract extraction	P
Eyes	192	23	
Patients	179	22	
Time to cataract extraction days, median (Q1, Q3)	-	455 (262, 780)	
Baseline VA letters, mean (SD)	62 (17)	56 (16)	0.09
6 months VA letters, mean (SD)	69 (14)	65 (11)	0.24
Crude VA change from baseline to 6 months letters, mean (95% CI)	+7 (5, 9)	+ 9 (2, 17)	0.38
Final VA, mean (SD)	65 (18)	73 (11)	**0.036**
Crude VA change at 36 months letters, mean (95% CI) [a]			
From baseline	+3 (0, 5)	+17 (8, 26)	**<0.01**
From 6 months	-4 (-6, -2)	+8 (2, 13)	**<0.01**
VA maintained from 6 months at 36 months (VA change ≥ -5 letters), %	66	91	**<0.01**
VA loss from 6 months at 36 months ≥ 10 letters, %	23	4	0.065
VA loss from 6 months at 36 months ≥ 15 letters, %	17	4	0.21

SD, standard deviation; Q1, first quartile; Q3, third quartile; CI, confidence interval; VA, visual acuity (logMAR letters).
[a] Last observation carried forward for non-completers.
Significant p-values are highlighted in bold.
